# Sparing the Brain, Saving the Breast: Thoracic Segmental Spinal Anesthesia in Frail Geriatric Patients

**DOI:** 10.7759/cureus.117579

**Published:** 2026-10-07

**Authors:** Paolo Scimia, Massimiliano Luca D'Agostino, Roberto Starnari, Luca Gentili, Antonio De Cato

**Affiliations:** 1 Intensive Care Unit, Department of Anesthesia, Giuseppe Mazzini Hospital, Teramo, ITA; 2 Department of Clinical Medicine, Public Health and Life Science, University of L'Aquila, L'Aquila, ITA; 3 Department of Anesthesiology, Istituto Nazionale di Ricovero e Cura per Anziani (INRCA), Ancona, ITA; 4 Department of Anesthesia and Intensive Care Unit, Santa Maria Goretti Hospital, Latina, ITA

**Keywords:** breast surgery, elderly frail patients, regional anesthesia, ropivacaine, spontaneous ventilation, thoracic segmental spinal anesthesia

## Abstract

Thoracic segmental spinal anesthesia (TSSA) provides a selective neuraxial block while preserving spontaneous ventilation and may therefore be useful in frail older patients in whom general anesthesia carries increased cardiorespiratory and neurocognitive risk. We describe three women aged 81, 91, and 99 years (American Society of Anesthesiologists (ASA) physical status III, IV, and III, respectively) who underwent breast quadrantectomy, with sentinel lymph node biopsy in two cases, under TSSA as the sole anesthetic technique. After informed discussion of the individual risks and benefits, spinal anesthesia was performed at the T7-T8 interspace using a 25-gauge Whitacre needle (Becton, Dickinson and Company (BD), Franklin Lakes, New Jersey, USA). Hypobaric ropivacaine was administered at a low dose (7.5 mg in two patients and 6 mg in one), without intrathecal adjuvants or intraoperative sedation. All procedures were completed without conversion to general anesthesia or additional anesthetic supplementation. Spontaneous ventilation was maintained throughout, and no clinically relevant hemodynamic, respiratory, or neurological complications were observed. Lower-limb motor function was preserved at the end of surgery, and scheduled intravenous paracetamol alone provided postoperative analgesia during the first 24 hours. Scores on the Italian-validated Quality of Recovery-15 (QoR-15I) questionnaire remained high at 24 and 48 hours. These cases suggest that low-dose TSSA may be a feasible anesthetic option for selected frail elderly patients undergoing breast surgery. Larger prospective studies are required to better establish its safety, indications, and reproducibility.

## Introduction

The elderly frail patient is characterized by reduced physiological functionality, affecting both the neurocognitive and cardiorespiratory domains [1,2]. Frailty is a state of decreased homeostatic reserve that increases vulnerability to even modest perioperative stressors, and its prevalence rises steeply with age, reaching approximately one-quarter of patients older than 85 years [3]. In this population, general anesthesia (GA) is not a physiologically neutral intervention. Airway instrumentation, positive-pressure ventilation, and systemic exposure to hypnotics, neuromuscular blocking agents, and opioids can meaningfully worsen an already limited cardiorespiratory reserve, translating into a higher risk of prolonged or difficult ventilator weaning and postoperative pulmonary complications. Moreover, older frail patients undergoing GA are at increased risk of postoperative delirium and postoperative cognitive dysfunction, both of which carry independent associations with increased morbidity, prolonged hospitalization, functional decline, and mortality in older surgical patients [4,5].

Regional anesthesia has therefore been advocated as a means of reducing this dual burden. Although thoracic paravertebral block is currently considered the gold standard technique for analgesia in breast surgery, its proven efficacy is coupled with inherent risks and the requirement for advanced technical skills. Consequently, recent scientific research has focused heavily on identifying alternative regional options that can offer comparable analgesic efficacy while ensuring an improved safety profile. Among these approaches, fascial plane blocks of the chest wall--including interpectoral, pectoserratus, serratus anterior plane, and erector spinae plane blocks--are effective techniques for perioperative analgesia in breast surgery [6]. However, in our view, fascial plane blocks present some practical limitations when considered for this alternative purpose in the frail elderly patient: they typically require relatively large volumes of local anesthetic to achieve adequate spread across multiple fascial planes, which raises the theoretical risk of local anesthetic systemic toxicity (LAST), especially when repeated or combined injections are needed to cover the entire surgical field of a quadrantectomy with axillary or sentinel node involvement. Furthermore, because they provide analgesia rather than complete surgical anesthesia, they are generally combined with moderate-to-deep intraoperative sedation to ensure patient comfort and immobility, only partially avoiding the neurocognitive and respiratory concerns that a fully awake technique aims to prevent. Moreover, despite their widespread adoption, fundamental questions regarding the optimal dosing, mechanisms of action, and overall efficacy and safety profile of fascial plane blocks remain largely unresolved [7].

In light of this, thoracic segmental spinal anesthesia (TSSA) represents a different paradigm. By injecting a small dose of local anesthetic directly at a thoracic interspace, this technique produces a selective sensory and sympathetic block confined to the metameres required for surgery, while sparing the lower limbs and without requiring any sedative or hypnotic drug [8,9]. Its clinical feasibility was demonstrated by van Zundert and colleagues in a patient with severe lung disease undergoing laparoscopic cholecystectomy [10], and subsequent magnetic resonance imaging studies clarified that the thoracic subarachnoid space is wider than traditionally assumed, providing a reassuring anatomical margin for cautious thoracic puncture when performed by experienced operators [11]. TSSA has since been described across a broad range of surgical specialties, including breast and axillary surgery [12], and recent narrative reviews and consensus documents have proposed structured "bundles" and safety checklists to standardize its use [1,2,8,9].

## Case presentation

All three patients underwent a preoperative assessment by a multidisciplinary team that included the anesthesiologist and the breast surgeon. After discussing the risk-benefit ratio with each patient, the neuraxial approach was determined to be preferable to GA. In addition, informed consent was obtained from each patient.

TSSA was performed with the patient in the sitting position, using the inferior angle of the scapula as a surface landmark corresponding to the T7 vertebral level, to guide identification of the T7-T8 interspace. A midline approach with a 25-gauge Whitacre needle (Becton, Dickinson and Company (BD), Franklin Lakes, New Jersey, USA) was used in a single-shot technique under strict aseptic conditions (skin antisepsis, sterile drapes, and a sterile gown, cap, mask, and gloves for the operator) to achieve a selective segmental block (Figure 1). After injection, the patient was positioned in the lateral decubitus position with the side to be operated on uppermost for six to seven minutes to promote a selective, unilateral spread of the block, and was then turned to the supine position.

**Figure 1:**
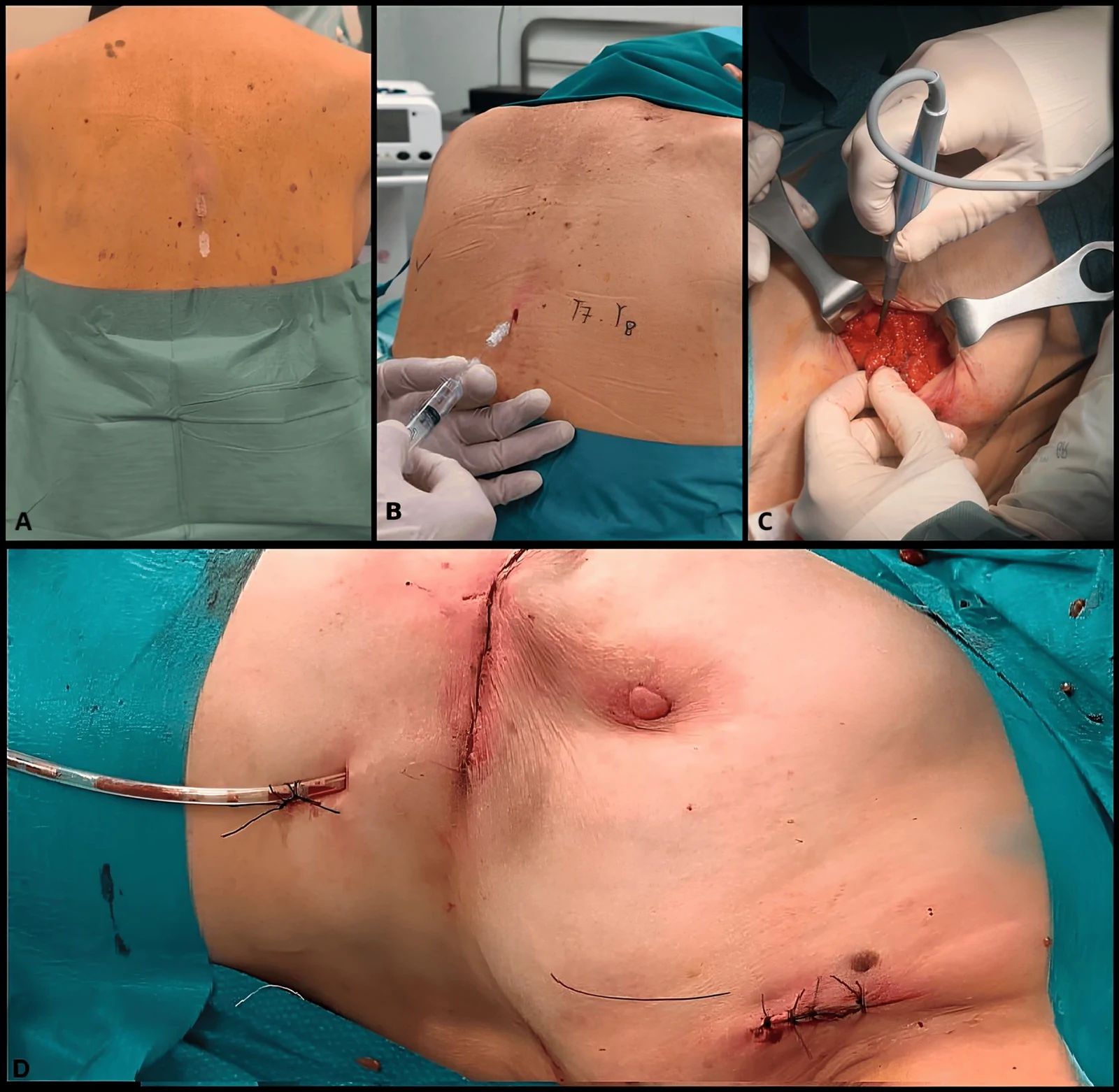
Representative images from cases managed with thoracic segmental spinal anesthesia. (A) Patient in the sitting position after skin preparation, with identification of the thoracic midline and visualization of a drop of cerebrospinal fluid confirming correct needle placement. (B) Identification of the T7-T8 interspace, followed by injection of local anesthetic into the subarachnoid space. (C) Intraoperative view of breast quadrantectomy performed under thoracic segmental spinal anesthesia. (D) Postoperative appearance of the surgical site with drain placement.

Sensory block height was assessed bilaterally before skin incision and repeatedly during surgery, using a cold-warm discrimination test (iced and warmed containers) to assess loss of thermal sensation and a pinprick test with a blunt 27-gauge needle to identify the dermatomal extent of loss of sharp pain sensation. Surgery was allowed to start only after a dense sensory block covering the dermatomes of the surgical field had been confirmed.

A rescue strategy was defined in advance. In case of a patchy block or incomplete coverage at the periphery of the surgical field, local infiltration by the surgeon with a short-acting local anesthetic was planned to supplement the block; in case of major or complete block failure, prompt conversion to general anesthesia with a laryngeal mask airway or tracheal intubation was planned. Local infiltration was not used as a routine baseline supplement, so that the efficacy of TSSA alone could be evaluated, and neither rescue option was required in any of the three cases.

No periprocedural complications occurred in any of the three cases (no bloody tap, no paresthesia, no difficulty in identifying the subarachnoid space, and no post-dural puncture headache). Standard intraoperative monitoring included continuous electrocardiography (ECG) and pulse oximetry (SpO_2_) in all patients; blood pressure was monitored non-invasively in patient 1, whereas an invasive arterial line was used in patients 2 and 3, reflecting their higher cardiovascular risk profile (American Society of Anesthesiologists (ASA) IV status and extreme age, respectively). No sedative or hypnotic drug was administered in any case: the Richmond Agitation-Sedation Scale (RASS) score remained at 0 throughout the procedure [13], and spontaneous ventilation was preserved without any need for respiratory support. At the end of surgery, the Bromage score was 0 in all three patients [14], indicating no residual lower-limb motor block. The Italian-validated version of the Quality of Recovery-15 (QoR-15I) questionnaire [15] was administered at 24 and 48 hours postoperatively in all three patients, yielding identical scores at both time points in each patient: 148 in patient 1, 146 in patient 2, and 150 in patient 3. Postoperative analgesia in the first 24 hours was fully achieved with scheduled intravenous paracetamol 1 g every eight hours in all cases, without any requirement for opioids, non-steroidal anti-inflammatory drugs, or rescue analgesia. Patient 1 was discharged on postoperative day 3 (POD3), while patients 2 and 3 were discharged on postoperative day 4 (POD4). A clinical assessment performed on postoperative day 3 in all three patients--coinciding with discharge in patient 1 and preceding discharge by one day in patients 2 and 3--did not identify any neurological or cardiorespiratory complications. The main clinical and anesthetic features of the three cases are summarized in Table 1.

**Table 1:** Main clinical, anesthetic, and outcome data of the patients. BMI: body mass index; ASA: American Society of Anesthesiologists; ECG: electrocardiography; SpO_2_: peripheral oxygen saturation; RASS: Richmond Agitation-Sedation Scale; QoR-15I: Italian-validated version of the Quality of Recovery-15 questionnaire (score range 0-150, higher scores indicating better recovery); POD: postoperative day.

Variable	Patient 1	Patient 2	Patient 3
Age (years)	81	91	99
Height/weight	150 cm/60 kg	152 cm/48 kg	152 cm/46 kg
BMI (kg/m²)	26.7	20.8	19.9
ASA physical status	III	IV	III
Main comorbidities	Type 2 diabetes mellitus, hypertensive heart disease, osteoporosis, anxious-depressive syndrome	Hypertensive heart disease, hyperhomocysteinemia, carotid atheromatosis, dyslipidemia	Hypertensive heart disease, first-degree atrioventricular block, early cognitive decline
Surgical procedure	Quadrantectomy + sentinel lymph node biopsy, left breast	Quadrantectomy + sentinel lymph node biopsy, right breast	Quadrantectomy, right breast (no sentinel node biopsy)
Puncture level/position	T7-T8, sitting position, inferior scapular angle landmark	T7-T8, sitting position, inferior scapular angle landmark	T7-T8, sitting position, inferior scapular angle landmark
Needle/technique	25G Whitacre, single-shot	25G Whitacre, single-shot	25G Whitacre, single-shot
Local anesthetic	Hypobaric ropivacaine 0.25%, 7.5 mg	Hypobaric ropivacaine 0.25%, 7.5 mg	Hypobaric ropivacaine 0.24%, 6 mg
Intrathecal adjuvants	None	None	None
Sedation/RASS	None/RASS 0	None/RASS 0	None/RASS 0
Hemodynamic monitoring	ECG, SpO_2_, non-invasive blood pressure	ECG, SpO_2_, invasive arterial pressure	ECG, SpO_2_, invasive arterial pressure
Periprocedural complications	None	None	None
Bromage score (end of surgery)	0	0	0
QoR-15I (24 h/48 h)	148/148	146/146	150/150
Postoperative analgesia (0-24 h)	Paracetamol 1 g/8 h only	Paracetamol 1 g/8 h only	Paracetamol 1 g/8 h only
Hospital discharge	POD3	POD4	POD4
Neurological/cardiorespiratory complications (POD3 assessment)	None	None	None

Case 1

An 81-year-old woman (BMI 26.7 kg/m², ASA III) with type 2 diabetes mellitus, hypertensive heart disease, osteoporosis, and an anxious-depressive syndrome was scheduled for quadrantectomy with sentinel lymph node biopsy of the left breast. Given her multiple comorbidities, avoidance of sedative and hypnotic drugs was considered clinically desirable. TSSA was performed according to the standardized technique described above, using hypobaric ropivacaine 0.25% (7.5 mg) and providing adequate surgical anesthesia without need for supplementation; blood pressure was monitored non-invasively, consistent with her comparatively lower cardiovascular risk relative to the other two patients, and no vasopressor was required. The QoR-15I yielded identical scores of 148 at both 24 and 48 hours postoperatively.

Case 2

A 91-year-old woman (BMI 20.8 kg/m², ASA IV) with hypertensive heart disease, dyslipidemia, hyperhomocysteinemia, and carotid atheromatosis was scheduled for quadrantectomy with sentinel lymph node biopsy of the right breast. Considering her advanced age and elevated anesthesiological risk, a neuraxial strategy avoiding airway manipulation and systemic sedative exposure was considered particularly appropriate. TSSA was performed according to the standardized technique described above, using hypobaric ropivacaine 0.25% (7.5 mg) and providing complete surgical anesthesia without the need for supplementation; given her ASA IV status, an invasive arterial line was used for continuous blood pressure monitoring, allowing beat-to-beat surveillance, and no vasopressor was required. The QoR-15I scores were identical at 24 and 48 hours postoperatively, with a score of 146 at both time points.

Case 3

A 99-year-old woman (BMI 19.9 kg/m², ASA III) with hypertensive heart disease, first-degree atrioventricular block, and early cognitive decline was scheduled for quadrantectomy of the right breast without sentinel lymph node biopsy. Given her extreme age and cardiac comorbidities, a neuraxial strategy was considered particularly appropriate. TSSA was performed according to the standardized technique described above, using hypobaric ropivacaine 0.24% (6 mg) and providing adequate surgical anesthesia without the need for supplementation; as in patient 2, an invasive arterial line was used for continuous blood pressure monitoring given the patient's age and cardiac conduction abnormality, with no vasopressor requirement and no arrhythmic events. The QoR-15I score was 150 at both 24 and 48 hours postoperatively.

## Discussion

This three-case series illustrates the feasibility of selective TSSA, using low-concentration hypobaric ropivacaine without intrathecal adjuvants, as the sole anesthetic technique for breast quadrantectomy in elderly frail patients. In all three patients, a single-shot injection at the T7-T8 level provided a selective segmental block adequate for the surgical field without local infiltration, systemic supplementation, or conversion to another technique, despite advanced age (81 to 99 years) and elevated anesthesiological risk (ASA III-IV). By utilizing a reduced dose of low-concentration ropivacaine, selective TSSA achieves an effective differential sensory block. This limits anesthesia strictly to the operative thoracic dermatomes while preserving diaphragmatic motor function. The block was intended to cover the dermatomes required for breast surgery, namely T2-T6: the skin and parenchyma of the breast are innervated mainly by the anterior and lateral cutaneous branches of the second to sixth intercostal nerves, while the axilla, involved in sentinel lymph node biopsy, is supplied largely by the intercostobrachial nerve (T2). The supraclavicular nerves (C3-C4) also contribute to the sensory innervation of the upper chest wall; this cervical territory is not expected to be reached by a low-dose mid-thoracic injection, which is consistent with the preservation of phrenic (C3-C5) and diaphragmatic function, and any discomfort arising from it would have been managed according to the rescue strategy described above. The reduced local anesthetic concentrations and doses used (0.24-0.25% ropivacaine, 6-7.5 mg) were chosen to limit cephalad and sympathetic spread, and no patient required vasopressor support or developed clinically relevant hypotension or bradycardia, including patient 3, who had a first-degree atrioventricular block. This does not imply that sympathetic block was absent: a segmental sympathetic block, which was not specifically quantified, was presumably present within the blocked thoracic metameres, but it had no clinically relevant hemodynamic consequences. With a low dose of local anesthetic injected at a mid-thoracic interspace, cephalad and caudal spread is restricted, and the extensive sympathectomy typical of conventional lumbar spinal anesthesia does not occur: the lower thoracic and lumbar sympathetic outflow, which controls a large part of the splanchnic and lower-limb vascular capacitance, is expected to be largely spared, thereby preserving venous return and compensatory vasoconstriction. Any involvement of the cardioaccelerator fibers (T1-T4) was not clinically apparent, as no bradycardia was observed. A Bromage score of 0 at the end of surgery in all three patients confirmed preserved lower-limb motor function despite adequate sensory-sympathetic block, a differential profile favored by the low ropivacaine concentration used; in frail elderly patients, even transient motor weakness can delay mobilization and increase the risk of deconditioning, venous thromboembolism, and delirium related to bed rest. Recovery quality, assessed with the QoR-15I [15], remained unchanged between 24 and 48 hours in each patient (148, 146, and 150 in patients 1, 2, and 3, respectively). Pain control in the first 24 postoperative hours was achieved with scheduled paracetamol alone in all three cases, without opioids, non-steroidal anti-inflammatory drugs, or rescue analgesia, an opioid-sparing profile that is particularly advantageous in frail older patients prone to opioid-related respiratory depression, constipation, nausea, and delirium.

The 2026 PROSPECT guidelines for oncological breast surgery focus on postoperative pain management. They recommend single-shot fascial plane blocks--erector spinae plane, interpectoral, pectoserratus, and serratus anterior plane--as part of a multimodal analgesic strategy [6]. These guidelines do not address fascial plane blocks as a primary anesthetic technique, and they make no comparison with TSSA. In our view, using them for this alternative purpose carries practical limitations in frail elderly patients. Covering a quadrantectomy extending toward the axilla may require large volumes or multiple blocks, raising a theoretical risk of LAST. Because these blocks provide analgesia rather than complete surgical anesthesia, they are typically combined with sedation or light GA for patient comfort. These points reflect our interpretation of the literature, not a PROSPECT conclusion. TSSA and fascial plane blocks are not necessarily antagonistic; they may be complementary, chosen according to patient risk, extent of surgery, and operator expertise.

This flexibility in technique selection was mirrored in our approach to drug selection. Using a single agent, ropivacaine, at a fixed low concentration (0.24-0.25%) and modest dose (6-7.5 mg), administered as a hypobaric solution without intrathecal adjuvants, was intended to maximize block predictability while minimizing adjuvant-related side effects such as pruritus, urinary retention, and delayed respiratory depression. The use of low-concentration hypobaric ropivacaine without intrathecal adjuvants was intended to achieve an adequate segmental block while limiting motor and sympathetic involvement. The choice of a hypobaric solution combined with precise T7-T8 puncture allowed controlled, limited cephalad spread, consistent with evidence that baricity selection can modulate block spread and side-effect profile in thoracic spinal anesthesia [16]. Similar individualized, low-dose TSSA protocols have been reported in other frail or high-risk surgical scenarios, including emergency laparoscopic management of an acute abdominal emergency in a patient with advanced systemic sclerosis [17], emergency colostomy in a patient with Turner syndrome [18], and a series of high-risk patients across different surgical specialties [19]. These reports, together with our three cases, show that low-dose TSSA has been used in several high-risk clinical settings. However, the available evidence remains largely limited to case reports and small case series.

The main safety concern with thoracic subarachnoid puncture is direct needle injury to the spinal cord, particularly to the dorsal columns, which form the posterior aspect of the cord and are therefore the first neural structures along the needle path. Although the risk of permanent neurological sequelae appears to be extremely low when a rigorous, standardized technique is applied by experienced operators, it cannot be considered negligible. Anatomical and imaging data provide a rationale for a safety margin at mid-thoracic levels. In a magnetic resonance imaging study, the spinal cord tended to follow the straightest course through the kyphotic thoracic canal, and the posterior dura-spinal cord distance was significantly greater in the mid-thoracic region than at upper and lower thoracic levels (e.g., 9.5 mm at T6 vs. 4.7 mm at T1 and 3.7 mm at T12) [11]. In addition, the marked caudal angulation of the mid-thoracic spinous processes imposes a steep needle entry angle, so that the needle crosses the posterior subarachnoid space obliquely, further increasing its distance from the cord [11,20]. Consistent with these observations, in a retrospective series of 1,406 patients receiving thoracic spinal anesthesia at the T8-T11 interspaces with 27-gauge needles, paresthesia occurred in 5.9% of cases, all transient and resolving within three days, and no permanent neurological sequelae were observed [20]. Nevertheless, the absence of events in 1,406 patients is still compatible with an incidence of up to approximately 0.2% (upper limit of the 95% confidence interval estimated with the rule of three); this rare but potentially devastating complication therefore cannot be excluded, and the evidence supporting the neurological safety of TSSA remains limited. In our cases, a midline approach with a 25-gauge pencil-point needle was used, and the absence of any sedation allowed the patients to report paresthesia immediately during needle advancement; no paresthesia occurred in any of the three patients.

Beyond the technical aspects of the block, the written informed consent of all three patients documented an explicit shared discussion of the individual risk-benefit ratio leading to the choice of a neuraxial strategy, an element of patient-centered anesthetic planning that we consider particularly relevant in the frail elderly population, where the perceived burden of general anesthesia and patient preference should be incorporated into decision-making alongside clinical risk stratification. Clinically, patient 1 was discharged on postoperative day 3, while patients 2 and 3, who were older and had a higher burden of comorbidity, were discharged on postoperative day 4; no neurological or cardiorespiratory complications were identified at the postoperative day 3 assessment or during the remainder of hospitalization in any patient.

Limitations

These observations should be interpreted in light of several limitations. This is a small and selected case series without a control group, so no conclusions can be drawn regarding the superiority of TSSA over fascial plane blocks or general anesthesia; the favorable outcomes may reflect careful patient selection, meticulous technique, and close multidisciplinary collaboration rather than an inherent property of the technique itself. Larger, comparative, prospective studies are needed to confirm the reproducibility of these findings and to better define the indications, safety profile, and long-term outcomes of selective TSSA in elderly frail patients undergoing breast surgery.

## Conclusions

Selective thoracic segmental spinal anesthesia provided adequate surgical anesthesia for breast quadrantectomy in the three elderly frail patients described in this series. Low-dose hypobaric ropivacaine administered at the T7-T8 level allowed surgery to be completed without sedation, airway intervention, or conversion to general anesthesia, while preserving spontaneous ventilation and lower-limb motor function. Although these observations are encouraging, they derive from a small, selected case series and should not be interpreted as evidence of superiority over general anesthesia or other regional techniques.

Taken together, these findings suggest that selective TSSA may represent a useful alternative strategy in the anesthesiologist's armamentarium for breast surgery in frail elderly patients. Larger prospective studies are needed to confirm its reproducibility and broader applicability, as well as to better define patient selection criteria and the clinical settings in which this technique may offer the greatest benefit. In light of the promising results we have directly observed, we believe in the potential of this approach, and we hope that other centers will also appreciate the advantages of this technique and adopt it in carefully selected cases.
